# Paper-Based Colorimetric Detection of miRNA-21 Using Pre-Activated Nylon Membrane and Peroxidase-Mimetic Activity of Cysteamine-Capped Gold Nanoparticles

**DOI:** 10.3390/bios13010074

**Published:** 2023-01-01

**Authors:** Maliana El Aamri, Hasna Mohammadi, Aziz Amine

**Affiliations:** Chemical Analysis and Biosensors Research Group, Laboratory of Process Engineering & Environment, Faculty of Sciences and Techniques, Hassan II University of Casablanca, P.A. 146, Mohammedia 28806, Morocco

**Keywords:** colorimetric detection, nanozyme, nylon paper, microRNA, genosensors, gold nanoparticles, smartphone

## Abstract

Irregular expression of MicroRNA-21 (miRNA-21) is considered as a promising biomarker for early cancer diagnosis. In this paper, a new genosensor based on paper and nanozyme activity of cysteamine-capped gold nanoparticles (Cys/AuNPs) was developed to detect picomolar concentrations of miRNA-21. Such nanozyme catalyzes the colorimetric reaction of hydrogen peroxide (H_2_O_2_) and 3,3′,5,5′ tetramethylbenzidine (TMB), to produce a blue color measurable by a smartphone. Due to their positive charge, Cys/AuNPs were attached to the negative phosphate groups of the DNA strand backbone via electrostatic interactions, leading to the quantitative determination of miRNA-21 concentration by the peroxidase-like activity of Cys/AuNPs. Furthermore, a paper-based assay was carried out on nylon disk devices to allow fast immobilization of DNA_probe_. After performing the paper-based assay, a good linear range was observed between 1 pM and 1 nM (Y = 0.080 [MiRNA-21]/pM + 13.846, R^2^ = 0.993) with a detection limit of 0.5 pM. The developed method was effective, selective, and sensitive for the miRNA-21 detection. The application of the proposed method for miRNA-21 detection was examined in a human serum sample, and a recovery rate of 90.0–97.6% was obtained showing the acceptable accuracy of the developed approach.

## 1. Introduction

MicroRNAs (miRNAs or miR) are noncoding RNAs that include 19–23 nucleotides and affect gene expression as well as a number of physiological mechanisms such as proliferation, cell differentiation, apoptosis, and immunity. Abnormal expression of certain miRNAs has been linked to a variety of diseases, mainly cancer. [1,2,3,4]. MiRNA-21 is an example of a gene that is overexpressed in various cancer tissues, including breast cancer, prostate cancer, gastric cancer, and lung cancers. Consequently, quantitative analyses of miRNA-21 can be used as biomarkers in the early diagnosis of cancer [5,6,7].

MiRNA-21 detection is challenging due to its unique characteristics, which include the low concentration in sample total RNA, short length, and high sequence similarity among family members. However, the emergence of biosensing approaches such as luminescence sensors, electrochemical biosensors, and colorimetric sensors has resulted in the development of powerful approaches to detect miR-21 expression accurately and quantitatively [8,9,10,11,12,13]. Colorimetric sensors, for example, have recently attracted attention due to their uncomplicated application and fabrication, visual detection, and low cost [14]. Nevertheless, developing colorimetric tests with the same sensitivity as electrochemical strategies remains an arduous mission. Many nanocomposites and nanoclusters with enzyme-mimicking activities have recently been discovered for the detection of diverse analytes, essentially proteins, DNA, and heavy metal ions [15,16,17,18,19,20].

Recently, with the fast development of nanotechnology, new biosensors based on various nanomaterials have been continuously explored to strengthen their analytical performance and their miRNAs detection. The investigated nanomaterials included graphene oxide [21], carbon black [22], carbon nanotube [23], molybdenum disulfide nanosheets [24], metal–organic frameworks [25], gold nanoparticles (AuNPs) [26], etc. They have not only exhibited high sensitivity and affinity for biomolecules but they have also protected bio-receptors from environmental harm, and improved diagnosis at the molecular and cellular levels. Moreover, gold nanoparticles have proved outstanding nanozyme-mimicking activity similar to that of peroxidase catalyzing the reaction of hydrogen peroxide (H_2_O_2_) and 3,3′,5,5′ tetramethylbenzidine (TMB) [27]. These gold nanoparticles’ catalytic activity was exploited in the present paper for miRNA-21 detection.

With the emergence of paper-based biosensors that leverage the advantages of micro-scale fluid physics, a new era of sensing has started in which paper-based biosensors minimize reagents, sample use, cost, test size, and overall automation [28]. Paper has been presented as a viable material for the development of microfluidic paper-based analytical strategies. Until now, a variety of processing methods have been presented to develop paper-based detection by designing hydrophilic/hydrophobic microstructures on paper substrates, mainly paper cutting [29], wax printing [30], wax dipping [31], plotting [32], inkjet printing [33], plasma treatment [34], and photolithography [35]. Paper-based biosensors provide several unique benefits, namely, low cost, simplicity of construction, robust capillarity, and strong biological compatibility for applications in clinical screening, food quality control, and environmental monitoring.

Colorimetric detection allows the use of a smartphone, which has recently gained popularity as a biomedical sensing instrument because of its high accessibility, user-friendliness, and increasing processing capacity. They are a viable option to replace traditional laboratory testing devices, which are usually bulky, expensive, and often require separate computers and qualified personnel for the operation. However, mobile applications opened directly through a smartphone can execute and automate much of the data collection and processing [36].

In this paper, the colorimetric detection for miR-21 as a cancer biomarker was carried out on paper. An unmodified DNA_probe_ has been immobilized on a pre-activated nylon membrane via a cross-linker which is glutaraldehyde. For the first time, a positive cysteamine-capped gold nanoparticles (Cys/AuNPs) nanozyme indicator and a nylon paper disk solid substrate were used in this work for the development of a colorimetric biosensor combined with a smartphone. This set allows on-site operations with reduced equipment. In the presence of the target sequence, a double strand was formed and the Cys/AuNPs were attached to the formed DNA–RNA hybrids via electrostatic interactions between the positive Cys/AuNPs and the negative phosphate groups of the DNA backbone. Therefore, the more miRNAs were present, the more positive Cys/AuNPs were attached. In this work, the revelation of the miRNA-21 concentration was performed by colorimetric substrate oxidation measurements using a smartphone camera. The data were treated with image J software. Spiked miRNA-21 was detected in human serum samples using the suggested colorimetric analytical approach, affirming that the proposed paper-based genosensor might be applied in bioanalysis.

## 2. Materials and Methods

### 2.1. Chemicals and Reagents

Pre-activated nylon paper immunodyne was purchased from Pall, France. Glutaraldehyde (Glu), chloroauric acid (HAuCl_4_), trisodium citrate dihydrate, and cysteamine (Cys) were bought from Merck, Darmstadt, Germany. Bovine serum albumin (BSA) as a blocking agent was obtained from Amresco Kerry, Ireland. A punch (Deli) of 6 mm was obtained from a local market. Tween 20 was obtained from VWR life science AMRESCO, Kerry, Ireland. Hydrogen peroxide (H_2_O_2_)/3,3′,5,5′-tetramethylbenzidine (TMB) liquid substrate systems were purchased from Sigma-Aldrich, Darmstadt, Germany. The chemicals used for the preparation of phosphate buffer saline (0.01 M PBS containing 2.7 mM KCl and 137 mM NaCl, pH 7.4) were bought from Merck, Darmstadt, Germany. All the used reagents were of analytical grade.

PBS was used as a washing buffer.

Acetate buffer saline (ABS) 0.1 M pH 4 was used as the reaction solution of H_2_O_2_/TMB.

Eurofins Genomics provided HPLC pure oligonucleotides as a lyophilized powder, The Ulis, France. The sequences of oligonucleotides were listed in Table 1. The stock solutions of synthetic oligonucleotides were prepared in ultra-pure water, aliquoted, and stored at −20 °C.

### 2.2. Apparatus

An android smartphone from Samsung (Galaxy A50 2019, The Netherlands) was employed to take the photos of the discs for the colorimetric analysis. The smartphone camera had a resolution of 25.0 megapixels. Images were taken manually in a box, under constant lighting conditions, and at a fixed distance. Regarding the digital analysis, the photos were treated with Image J software version 1.52 V (NIH, USA). The red value was used to calculate the color intensities. On the other hand, the data analysis and graphing software were treated and designed by OriginPro8 V8.0951 (B951).

For the characterization of Cys/AuNPs by Ultraviolet-Visible (UV-Vis) spectroscopy, a UV-Vis double beam spectrophotometer JENWAY (6850) (London, UK) with a 1.0-cm matched cell was employed.

The Fourier-Transform Infrared (FTIR) spectroscopy was performed with an IRAffinity-1S spectrophotometer (SHIMADZU, Kyoto, Japan) in the 4000–500 cm^−1^ attenuated total reflectance mode.

### 2.3. Preparation of AuNPs

Prior to use, all glassware was rinsed in aqua regia (HCl:HNO_3_, 3:1), washed with distilled water, and then kiln-dried. AuNPs were synthesized using citrate reduction of HAuCl_4_. Shortly, 100 mL of HAuCl_4_ (0.506 mM) was heated to a boil under intense stirring, then 10 mL of citric acid trisodium salt dihydrate (19.4 mM) was quickly added. The solution color shifted from pale yellow to an uncolored solution, then to black, and lastly to a wine-red homogeneous solution. The solution was cooled to room temperature, then filtered through a syringe filter (0.22 µm) to remove all large particles. The obtained solution was kept at 4 °C in the dark until use. The UV-vis absorption spectrum presented a ʎ_max_ at 520 nm.

### 2.4. Preparation of Cys/AuNPs

The functionalization of synthesized AuNPs by Cysteamine was carried out as described by Georgia et al. [37]. Various ratios (*V*/*V*) of 0.500 mM of cysteamine prepared in distilled water and the stock solution of the synthesized AuNPs were mixed. Functionalization was performed in an Eppendorf of 1mL, by adding 50 µL of the stock solution of AuNPs and various volumes of cysteamine. The final volume was 1 mL.

After overnight incubation at 4 °C, the modified AuNPs were purified by eliminating the excess of the un-adsorbed cysteamine by 3 cycles of centrifugation. Indeed, 1 mL of the solution was first centrifuged at 15,000 rpm for 30 min. Then, the supernatant was carefully eliminated and 900 µL of distilled water was added. The obtained pellets were re-suspended in distilled water via sonication at room temperature for 1 min. Lastly, after removing the excess of cysteamine, the Cys/AuNPs were stored in the dark at 4 °C.

### 2.5. Paper-Based Genosensor

The analytical device fabrication on the nylon membranes was performed using cheap instruments. The nylon membrane was punched giving 6 mm disks using a punch. The type of used membrane is a nylon-activated immunodyne.

The immobilization of the unmodified DNA_probe_ requests a cross-linking agent. Herein, a solution of glutaraldehyde (3%) and an appropriate concentration of DNA_probe_ (in 0.01 M PBS containing 2.7 mM KCl and 137 mM NaCl) was added to the surface of the nylon disk, the DNA_probe_ was fixed via C=N covalent bonding formation at room temperature. After 45 min, the disks were washed with distilled water three times. Then, a solution of 3% BSA (prepared in PBS) was added to the disks for 15 min to block the non-specific sites. Afterward, three washes were required to remove BSA excess. The prepared DNA_probe_ modified nylon disks were kept at 4 °C until use. The prepared paper-based genosensor was suggested as a ready-to-use device for on-site and rapid microRNA-21 detection.

### 2.6. miRNA-21 Hybridization Process

The hybridization was carried out by adding 10 µL of various concentrations ranging from 1 pM to 1 nM (in 0.01 M PBS containing 2.7 mM KCl and 137 mM NaCl, pH 7.4) to each paper-based genosensor. Then, the disks were kept for one hour at RT and washed thrice using the washing buffer.

### 2.7. Smartphone-Based Colorimetric Detection

After the hybridization step, 10 µL of a specific concentration of prepared positive Cys/AuNPs was poured on each paper disk for 15 min at room temperature. Then, three washes with PBS containing 0.05% tween-20 were required to remove excess Cys/AuNPs.

A solution of H_2_O_2_/TMB (8 mM/5 mM) prepared in ABS 0.1 M pH 4 was added to each paper disk. A blue color appeared on the disk and was stabilized after 10 min.

The obtained colored discs were captured using previously optimized conditions in our earlier work [8]. The disks were placed in a dark box, then the smartphone capture was performed with flashlights-on. A distance of 12 cm between the paper and the smartphone was maintained. The intensities of RGB colors were quantified by drawing a circle in the colored region of the disk using ImageJ Software.

## 3. Results and Discussion

### 3.1. Study of Cys/AuNPs Composites as Nanozyme

There is a growing interest in the employment of AuNPs for various applications due to their peroxidase-like activity. In this work, we used cysteamine to modify the negative charge of the AuNPs, leading to a positive charge on the surface of Cys/AuNPs composites. These composites will be attached to the phosphate groups of the DNA backbone by electrostatic interactions.

**Figure 1:**
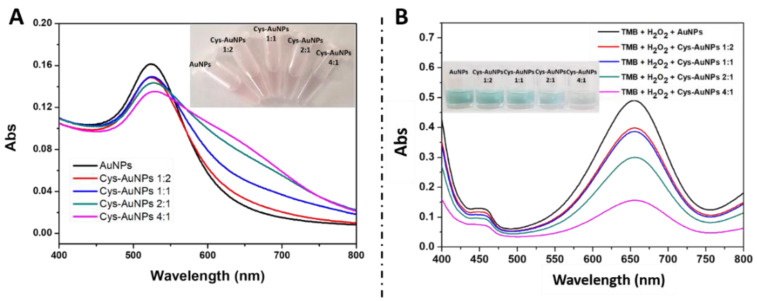
(**A**) Ultraviolet-Visible (UV-Vis) spectra for prepared cysteamine-capped gold nanoparticles (Cys/AuNPs) at various volume ratios of AuNPs and cysteamine. (**B**) UV-Vis spectra of 3,3′,5,5′-tetramethylbenzidine and hydrogen peroxide (TMB-H_2_O_2_) solution catalyzed by Cys/AuNPs composites at various volume ratios of AuNPs and cysteamine.

One of the major concerns about AuNPs is their tendency to aggregate in the presence of other substances causing negative effects on material performance. For this purpose, the volume ratios of the prepared AuNPs and cysteamine have been optimized. The obtained UV-Visible spectrums (Figure 1A) were similar to those obtained by Georgia et al. [37]. Figure 1A showed that the AuNPs aggregate by increasing the concentration of cysteamine. The obtained results were confirmed by UV-Visible spectra. Indeed, the absorbance decreases in the presence of a high volume of cysteamine explaining their aggregation, as indicated in the inset of Figure 1A. On the other hand, the mimic peroxidase activity of Cys/AuNPs was studied. As shown in Figure 1B, the peroxidase activity of Cys/AuNPs decreases as the amount of added cysteamine increases. Our results confirm those published by Lou-Franco et al. [38]. According to the obtained results in Figure 1A,B, a volume ratio of 1:2 of cysteamine (0.500 mM) and AuNPs (stock solution) was chosen as the optimum for the rest of the work. At this ratio, the AuNPs were sufficiently modified with cysteamine, nevertheless, retaining their peroxidase activity.

### 3.2. Characterization of the Nylon Membrane Disk before and after Immobilization of DNA_Probe_ and the Study of Electrostatic Interaction of DNA_probe_

In this study, we characterized the DNA_Probe_ immobilized on an activated nylon membrane disk using FTIR.

**Figure 2:**
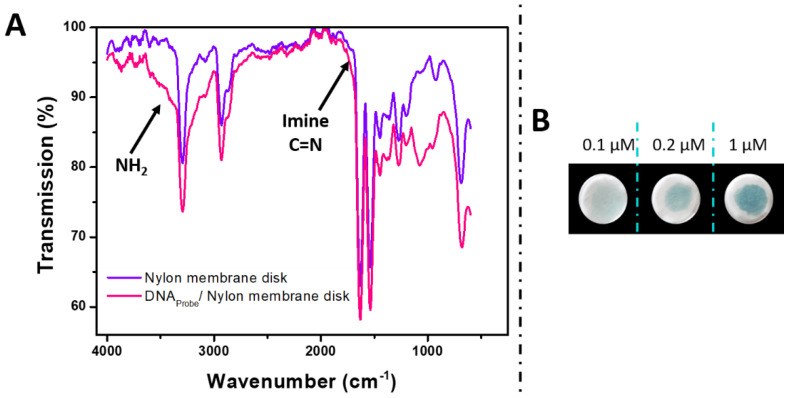
(**A**) FTIR spectra of immobilized DNA_probe_ on the nylon membrane disk. (**B**) TMB/H_2_O_2_ solution catalysis by Cys/AuNPs in the presence of different concentrations of DNA_probe_ immobilized on the nylon membrane.

In Figure 2A, the spectrum of the DNA_probe_ immobilized on the nylon disk reveals a large band overlapping with the nylon membrane spectrum from 3000 to 3600 cm^−1^. This band is characteristic of the NH_2_ of the DNA [39], confirming the presence of the DNA on the disk. On the same spectrum, a band at 1660 cm^−1^ overlapping with the nylon membrane band was observed after modification with DNA and glutaraldehyde. This band is attributed to the imine resulting from the interaction of the aldehyde and the amine groups of the membrane on one side and of the amine groups of the DNA on the other side [40] indicating a strong interaction between DNA and nylon disk via glutaraldehyde.

The attachment of Cys/AuNPs onto DNA strands depends on the concentration of DNA present on the surface. In this study, we confirmed that the attachment of these positively charged Cys/AuNPs depends on the concentration of single-stranded DNA immobilized on the nylon disk surface using an H_2_O_2_/TMB colorimetric assay (Figure 2B). Briefly, various concentrations of single-stranded DNA (0.1 µM, 0.2 µM and 1 µM) were immobilized on the nylon disk via glutaraldehyde as a cross-linker agent, then, a volume of the nanozyme was added to each disk. At last, the H_2_O_2_/TMB solution was added to each disk. The results showed that by increasing the concentration of DNA_probe_ on the surface, the intensity of the blue color increases. This is attributed to the higher attachment of the positively charged Cys/AuNPs composites to the DNA backbone’s negative phosphate groups through electrostatic interactions.

### 3.3. Optimization of Assay

In this work, the use of the smartphone is mainly due to its ability to detect small color variations on several surfaces. Furthermore, paper disks have been used as a solid substrate due to their excellent optical characteristics, low cost, and user-friendliness on-site.

An optimization was necessary to find the ideal conditions for the considered approach. In summary, using the developed DNA assay, different concentrations from 1 pM to 1 nM of miRNA-21 were prepared and incubated in the ready-to-use paper disks. Then, images from the colorimetric test were taken with a smartphone in a dark box with flashlights on and a distance of 12 cm conditions (parameters optimized in the previous work [8]), and the color intensities were automatically calculated by reading RGB values using Image J software.

Color intensities were plotted against miRNA-21 concentration as shown in Figure S1. The green and blue values did not show a noticeable change in color intensity when the concentration of miR-21 increased. In contrast, the red color proved to be sensitive to picture acquisition settings. A substantial correlation was notable between the red color intensity and miRNA-21 concentrations. Accordingly, the red channel was chosen as the best quantitative analytical parameter out of all investigated channels thanks to its high sensitivity to changes depending on miRNA-21 concentrations.

**Figure 3:**
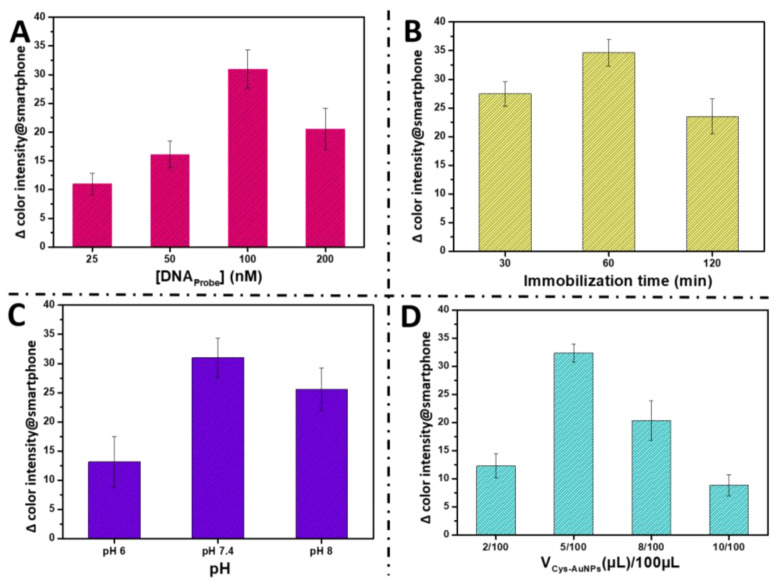
Optimization of factors impacting the paper-based miRNA-21 detection (**A**) DNA_probe_ concentration immobilized on paper disk, (**B**) immobilization time of DNA_probe_, (**C**) pH of immobilization step, and (**D**) dilution of prepared Cys/AuNPs nanozyme. The response of the paper-based genosensor developed before and after hybridization with 100 pM of miRNA-21. Three parallel experiments yielded error bars.

During experiments that aim to improve the sensitivity of the assay, various factors that may affect the signal were optimized, including the immobilization conditions of the DNA_probe_ and the nanozyme concentration used during the detection process. Herein, glutaraldehyde was used to immobilize the DNA_probe_. In order to enable a maximum hybridization, and as a consequence, to achieve a stable platform as well as a maximum signal intensity, the concentration of immobilized DNA probe was investigated. Accordingly, a concentration ranging from 25 to 200 nM of DNA_probe_ was studied and an optimum response was obtained using 100 nM of DNA_probe_ (Figure 3A). The immobilization time was also optimized using 30, 60, and 120 min and the optimum response was obtained within 60 min (Figure 3B). As a result, at high concentration of DNA_probe_ and after a long time of immobilization, the surface of the disk is strongly charged, resulting in good DNA_probe_ immobilization. However, this does not allow miRNA-21 hybridization because of the steric effect.

The pH is a critical factor in the immobilization of the DNA_probe_ on the pre-activated nylon membrane. In this framework, the immobilization buffer with several pHs was tested, namely pH 6, pH 7.4, and pH 8. Figure 3C showed that the signal was generally strong when pH 7.4 and 8 were used which is attributed to the nature of the pre-activated membrane. Finally, the concentration of the nanozyme Cys/AuNPs is also important in the developed approach. Therefore, 2 µL, 5 µL, 8 µL, and 10 µL of a prepared Cys/AuNPs suspension (as described in the Materials and Methods section) were added to distilled water for a total volume of 100 µL. Then, the obtained dilutions were used for the detection of miRNA-21. As shown in Figure 3D, the dilution of 5/100 revealed an excellent signal change, while at a higher concentration of positive Cys/AuNPs, the nanozyme was bound to the paper, even in the absence of the target.

### 3.4. Principle of the Developed Paper-Based Genosensor for the miRNA-21 Detection

**Scheme 1:**
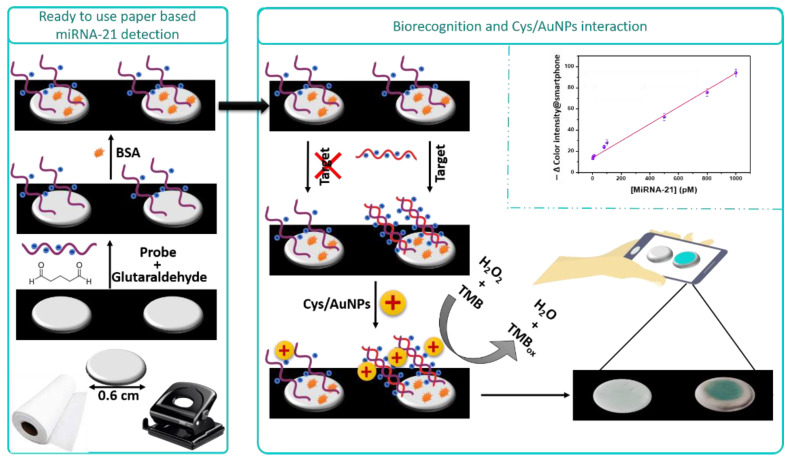
Illustration of paper-based colorimetric biosensor based on the electrostatic interaction of positive Cys/AuNPs and phosphate groups of DNA backbone and its use for miRNA-21 detection using a smartphone.

The analysis principle of the developed paper-based miRNA-21 detection is depicted in Scheme 1.

In this work, an activated nylon membrane surface was used as the solid substrate for the developed genosensor. Indeed, as shown in Scheme 1, a punch was used to construct disks of 6 mm in diameter. Afterward, the unmodified DNA_probe_ was immobilized on the disk using glutaraldehyde as a cross-linker. Then, BSA was used to block the non-specific sites. Eventually, a paper-based miRNA-21 detection was obtained and ready to use. In the presence of the target (miR-21), the DNA_probe_ was hybridized and DNA–RNA hybrids were formed. Then, the Cys/AuNPs composites were added. The positive Cys/AuNPs were attached to the negative phosphate groups of the hybrid backbones (DNA–RNA) via electrostatic interactions. The peroxidase-like activity of AuNPs as nanozyme catalysis the hydrogen peroxide and TMB reaction. Then, a blue color was produced. The intensity of the color increased with the increase in miRNA-21 concentrations. Afterward, each disk was captured by a smartphone camera in ideal lighting and distance conditions [8]. Finally, the color intensity of each disk was quantified using Image-J software.

### 3.5. Performance of the Paper-Based Genosensor

Properties of the proposed method based on the mimic peroxidase activity of Cys/AuNPs were investigated. A smartphone was used to measure the blue product of the catalytic reaction of H_2_O_2_/TMB in the presence of different concentrations of miRNA-21. 

**Figure 4:**
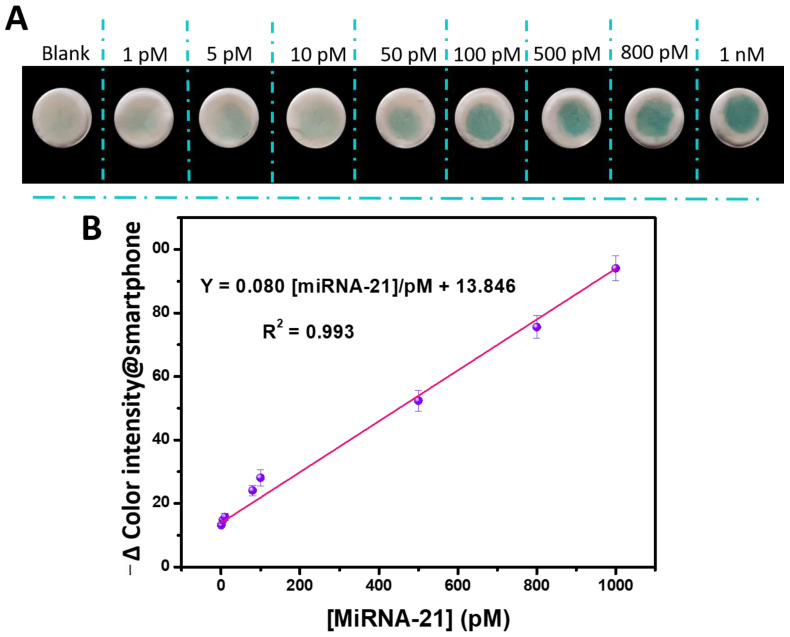
(**A**) The visual incremental color change observed for the paper-based genosensor ranging from 1 pM to 1 nM of miRNA-21. (**B**) Calibration plots of color intensities (I_target_ − I_blank_) against different miRNA-21 concentrations ranging from 1 pM to 1 nM. Three parallel experiments yielded error bars.

As shown in Figure 4A, color intensity increased gradually as the concentration of miRNA-21 increased from 1 pM to 1 nM. A linear range from 1 pM to 1 nM of miRNA-21 concentrations was obtained with a detection limit of 0.5 pM calculated using the 3 σ method (Figure 4B). The linear regression equation was Y = 0.080 [miRNA-21](pM) + 13.846 with an RSD of 4.6% for the slope of the calibration curve and an R^2^ = 0.993.

To evaluate the performance of the proposed sensor, Table 2 summarizes various colorimetric analytical approaches based on smartphone detection of miRNAs. The sensitivity of our analytical assay was comparable with those of other biosensors.

### 3.6. Selectivity and Stability Study of the Paper-Based Genosensor for the miRNA-21 Detection

**Figure 5:**
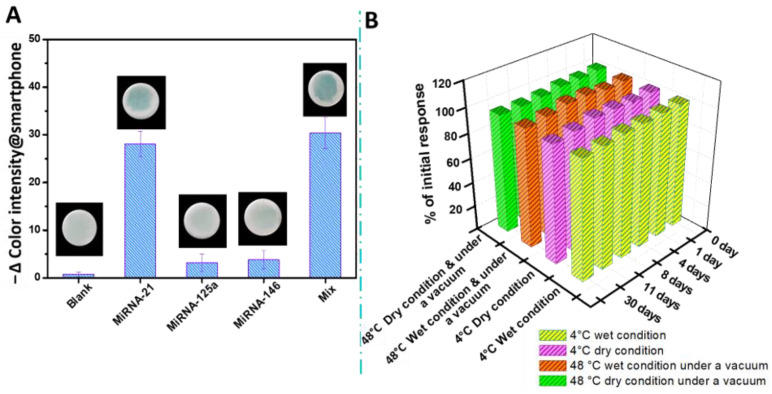
(**A**) Selectivity study toward noncomplementary miRNAs (miR-125a, miR-146), and a mixture of three microRNAs. (**B**) Stability study of the paper-based genosensor during various storage times (1 day, 4 days, 8 days, 11 days, and 30 days) in various storage conditions using 100 pM of the target. Three parallel experiments yielded error bars.

To further investigate the feasibility of the suggested genosensor, at the same concentration of miRNA-21 (100 pM), selectivity was tested for two non-complementary target miRNAs and mixed miRNA (100 pM). As Figure 5A shows, the intensity of the paper color toward miRNA-21 was considerably stronger than those toward non-complementary miRNAs, demonstrating excellent selectivity of the suggested approach.

Additionally, several storage conditions of the paper-based biosensor were examined. The bio-platform was first stored at a high temperature of 48 °C with and without PBS and under a vacuum. Meanwhile, other paper-based genosensors were kept with and without PBS at 4 °C. After 0, 4, 8, 11, and 30 days, the genosensor was tested by hybridization with 100 pM of miR-21. Figure 5B shows that even after 30 days at 4 °C and in wet and dry conditions, the constructed paper-based genosensor preserved around 95% of its initial response, making it suitable for practical usage. Even at exceptional conditions of 48 °C under vacuum, after 30 days, the developed paper-based miRNA-21 assay preserved around 96% of the initial response in wet and dry conditions.

### 3.7. Application of the Paper-Based Genosensor in Real Sample

To be useful in bioassays and practical applications, any genosensor should be able to endure the matrix effects of biological samples. Wherefore, to demonstrate the practical application of the present paper-based genosensor, miR-21 at various concentrations (10, 50, 500, and 1000 pM) were spiked into 20% human serum, to mimic the identical environment of real samples. Cys/AuNPs were added after the hybridization step. Then, the TMB and H_2_O_2_ were injected into the disks as described in the Materials and Methods section.

**Figure 6:**
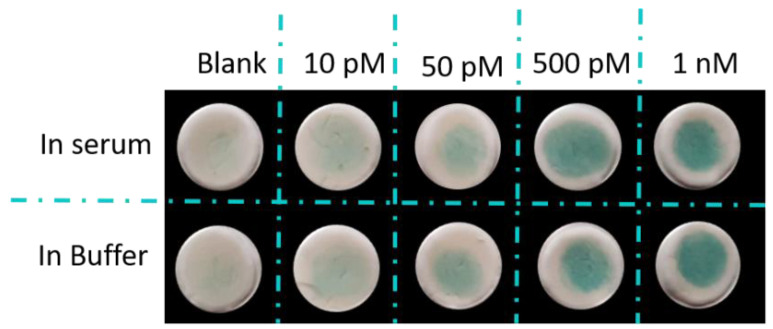
miRNA-21 detection in human serum samples and in PBS at different concentrations.

As shown in Figure 6 and Table 3, the obtained results are in concordance with those expected with a 90.0–97.6% recovery rate. Furthermore, the presence of interferants in the serum sample had no impact on our test method. Therefore, the suggested approach can be employed to quantify miRNA-21 in real samples.

## 4. Conclusions

In conclusion, a sensitive paper-based colorimetric biosensor was designed for the detection of miRNA-21. This innovative method was based on electrostatic interactions between positively charged Cys/AuNPs and negatively charged phosphate groups of the DNA–RNA hybrid formed on the pre-activated nylon membrane. The attached Cys/AuNPs nanozyme catalyzed the H_2_O_2_/TMB reaction to produce a blue color on the surface of the paper and was afterward measured by a smartphone. The intensity of the blue color depended on the amount of microRNA present in the sample. The combination of, first, the nylon disk as a mobile, stable, and ready-to-use platform, then, the Cys/AuNPs revelator of the presence of miRNA-21, and finally, a smartphone to read the signal allowed on-site quantitative testing. Using the present colorimetric method, microRNA-21 was detected at concentrations as low as 0.5 pM over a linear range of 1 pM to 1 nM. This approach has the potential to evaluate various DNA biomarkers.

## Figures and Tables

**Table 1:** Nucleic acids employed in the present work.

Nucleic Acid	Sequence (5′-3′)
Target miRNA-21	5′-UAGCUUAUCAGACUGAUGUUGA-3′
DNA_probe_ (complementary sequence of miRNA-21):	5′-AAATCAACATCAGTCTGATAAGCTA-3′
MiRNA-146 (non-complementary oligonucleotide)	5′-UGAGAACUGAAUUCCAUGGGUU-3’
MiRNA-125a (non-complementary oligonucleotide)	5′-UCCCUGAGACCCUUUAACCUGUGA-3’

**Table 2:** Comparative analyses of smartphone-based miRNA detection.

Strategies	Target	Solid Substrate	Regression Equation	Linear Range	Limit of Detection	Ref.
miRNA detection based on peroxidase mimetic activity of DNA-Ag/Pt NCs	miR-21	Whatman filter	y = 0.06x + 147.48	10–1000 pM	4.1 pM	[41]
miRNA detection based on silver NPs aggregation	miR-21	In solution	Y = 0.355 Logx − 0.067	1–300 nM	-	[36]
Paper-based sensor for miRNAs by combining GONET and the target-recycled signal amplification strategy	miR-122	Whatman filter	-	-	0.52 nM	[42]
miRNA detection based on the generation of molybdophosphate complex coupled with DNA hydrolysis	miR-21	96-well microplates	Y = 82.3 − 11.5 Logx	1–50,000 pM	0.6 pM	[8]
miRNA detection-based peroxidase-mimetic activity of cysteamine-capped gold nanoparticles	miR-21	Nylon paper	Y = 0.080x + 13.846	1–1000 nM	0.5 pM	This work

**Table 3:** Results of miRNA-21 detection in human serum samples using the suggested genosensor (n = 3).

Sample	Original [miR-21] (pM)	Spiked [miR-21] (pM)	Founded [miR-21] (pM)	Recovery (%)	RSD (%)
1	0	10.0	9.6	96.0	4.7
2	0	50.0	48.4	96.8	3.9
3	0	500.0	488.2	97.6	6.3
4	0	1000	900	90.0	7.2

## Supplementary Materials

The following supporting information can be downloaded at: https://www.mdpi.com/article/10.3390/bios13010074/s1, Figure S1. Relationship between the intensity of red, green and blue color values and the concentration of microRNA (1, 10, 100, and 1000 pM) in the proposed approach while exposed to flashlights-on in a dark box. Error bars were obtained from three parallel experiments.
