# Supplementary material for: Paper-Based Colorimetric Detection of miRNA-21 Using Pre-Activated Nylon Membrane and Peroxidase-Mimetic Activity of Cysteamine-Capped Gold Nanoparticles
Source: Biosensors (Basel). 2023 Jan 1;13(1):74. doi: 10.3390/bios13010074 (PMC9855695; doi:10.3390/bios13010074)
Supplement: Supplementary file 1 [file biosensors-13-00074-s001.zip › biosensors-1976428-supplementary.pdf]

biosensors MDPI

Supplementary material

Paper-Based Colorimetric Detection of MiRNA-21 Using Pre-activated Nylon Membrane and Peroxidase-Mimetic Activity of Cysteamine-Capped Gold Nanoparticles

Maliana El Aamri[1], Hasna Mohammadi[1], Aziz Amine[1*]

[1] Chemical Analysis and Biosensors Group, Laboratory of Process Engineering and Environment, Faculty of Sciences and Techniques, Hassan II University of Casablanca, B.P 146, Mohammedia, Morocco

*Corresponding authors: a.amine@univh2m.ac.ma (A. Amine)

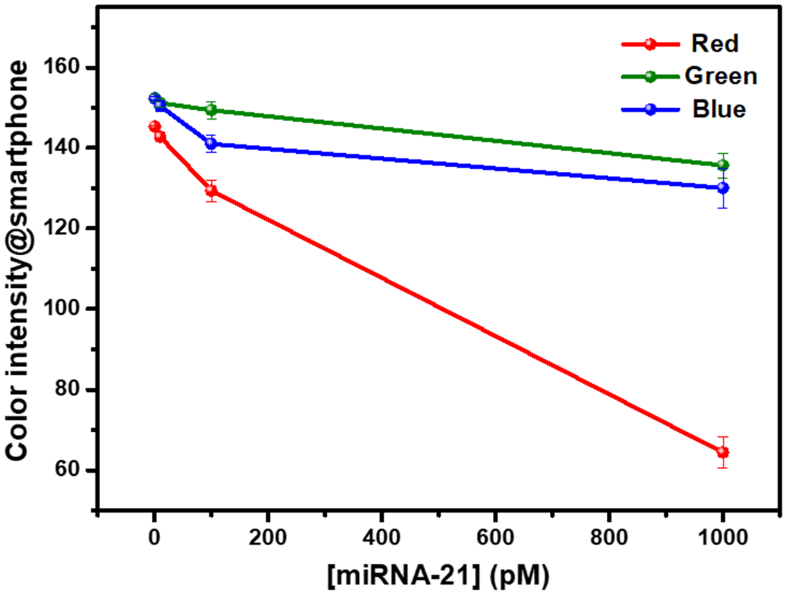

Figure S1. Relationship between the intensity of red, green, and blue color values and the concentration of microRNA (1, 10, 100, and 1000 pM) in the proposed approach while exposed to flash-lights-on in a dark box. Error bars were obtained from three parallel experiments.
